# Outcomes of localized corneal collagen crosslinking with a conventional device in progressive keratoconus

**DOI:** 10.1007/s00417-025-06803-y

**Published:** 2025-03-27

**Authors:** Ofri Vorobichik Berar, Rachel Shemesh, Nir Gomel, Yoav Berger, Irina S. Barequet

**Affiliations:** 1 Goldschleger Eye Institute, Faculty of Medical and Health Sciences, Sheba Medical Center, Tel Aviv University, Tel Hashomer, Israel; 2 Division of Ophthalmology, Faculty of Medical and Health Sciences, Tel-Aviv Medical Center, Tel Aviv University, Tel Aviv, Israel

**Keywords:** Keratoconus, Crosslinking, Localized crosslinking

## Abstract

**Purpose:**

To evaluate the outcomes of localized crosslinking (L-CXL) for progressive keratoconus utilizing a standard CXL device.

**Design:**

This retrospective cohort study.

**Methods:**

included patients diagnosed with progressive keratoconus and treated with a localized cone-centered CXL (based on corneal topography) using the accelerated CXL protocol with a standard CXL device.

**Results:**

We Identified 24consecutive eyes. The average BDVA before CXL was 0.282 ± 0.35 LogMar, remained overall stable at 12-month post-surgery at 0.204 ± 0.173 LogMar (*P* = 0.395). Ten eyes (of eight patients) (42%) demonstrated an improvement in BDVA at 12 months of 1–4 lines and none of the other eyes lost BDVA. These eyes had significantly lower pre-operative BDVA than the stable eyes (*P* = 0.034). Ten eyes (of eight patients) (42%) demonstrated an improvement in K-max at 12 months post-operatively, of at least 1D; six of these eyes had improvement in both BDVA and Kmax. None of the eyes developed an increase in Kmax throughout the follow-up.

**Conclusion:**

In this series, cone-centered L-CXL, using a conventional CXL device resulted in significant stabilization and even improvements in BDVA and Kmax in almost half of the eyes, without significant adverse events. Addressing the CXL application onto the affected area results in beneficial results.

## Abstract (highlights)

**Key messages:**

*****What is known***:**

- CXL is safe and effective in treating progressive keratoconus.
- Previous studies have shown that customized cone centered CXL is effective in treating keratoconus progression.

*****What is new***:**

- In this study, we proved the efficacy of the cone-centered L-CXL procedure using a standard CXL device. This is meaningful, especially nowadays when other more complex devices are not available.
- L-CXL even without eye tracking, is successful not only in stabilizing the keratoconus but even resulting in an improvement in BDVA and Kmax.
- The procedure is safe, with no evidence of progression in any of the eyes.

## Introduction

Keratoconus is a biomechanical weakness of the cornea, resulting in irregular corneal astigmatism that can progress and compromise vision [1]. Collagen crosslinking (CXL) is a well-established procedure for stopping progression of keratoconus and is considered the gold standard treatment for stabilizing progressive keratoconus in many countries [2–5]. In April 2016, the US Food and Drug Administration (FDA) approved Avedro's (Avedro, Inc, Waltham, MA) corneal CXL system for treatment of patients with progressive keratoconus and post-LASIK ectasia. Based on the early observation of improved visual acuity in some of the keratoconus eyes treated with CXL—Customized Localized CXL (L-CXL) was later proposed by Sinha Roy and Dupps [6]. They also provided proof of concept for patient-specific differential refractive responses to CXL. Using three-dimensional finite element models, they showed that smaller, focal, cone-centered CXL simulations provided the greatest topographic effects. Moreover, the risk of complications such as infection, haze, and stromal damage are also expected to diminish due to the decreased area of epithelial removal [6, 7]. This was examined in a few studies, demonstrating that CUSTOMIZED CXL, with eye tracking is as safe as the standard procedure, with stronger flattening in K-max and a faster epithelial healing period [8, 9]. Furthermore, because the stiffening in L-CXL is concentrated in the mechanically compromised area of the cornea, greater local topographical flattening is expected [6]. An emphasis on the need for eye tracking in the CXL system was mentioned in recent literature. Studies on advanced CXL systems with eye tracking capabilities have demonstrated that motion compensation can significantly affect UV beam delivery. An active eye tracking system, such as the Mosaic™ device (formerly by Avedro, Inc.), was designed to compensate for eye movement in real-time by adjusting the UV beam delivery.. The natural eye movements may result in degraded spatial localization, a decrease in the refractive impact, and nonuniformity in treatments [10]. An active eye tracking system, in which eye movement is compensated for by changes in the UV beam in real-time, such as the Mosaic device, was suggested to overcome these disadvantages and help avoid potential limbal stem cell damage and unwanted or diminished refractive effects., This system showed promise in delivering precise CUSTOMIZED CXL treatment. However, this device is no longer commercially available following corporate changes.. In this study, we attempt to assess the long-term outcomes of cone-centered L-CXL utilizing a standard CXL device with an accelerated protocol.

## Methods

### Study design

This retrospective study included consecutive patients diagnosed with progressive keratoconus previously treated by cone-centered L-CXL between 2018 and 2020. Institutional Review Board (IRB) approval was obtained, and the study adhered to the tenets of the Declaration of Helsinki. The diagnosis of progression was based on documented corneal tomographic changes compared to previous measurements. The definition of progression was defined as: > 1 D increase in the steepest radius of curvature of the anterior corneal surface (K-max); a decrease in minimal pachymetry of over 10 µ within one year.

### Imaging

All patients underwent tomographic imaging by trained technicians using the Sirius tomographer (CSO, Costruzione Strumenti Oftalmici, Florence, Italy), which combines Placido disk topography with Scheimpflug tomography of the anterior segment. Contact lens wearers were instructed to discontinue contact lens wear for at least 72 h for soft contact lenses and 2 weeks for rigid gas permeable lenses prior to examinations.

**Fig. 1:**
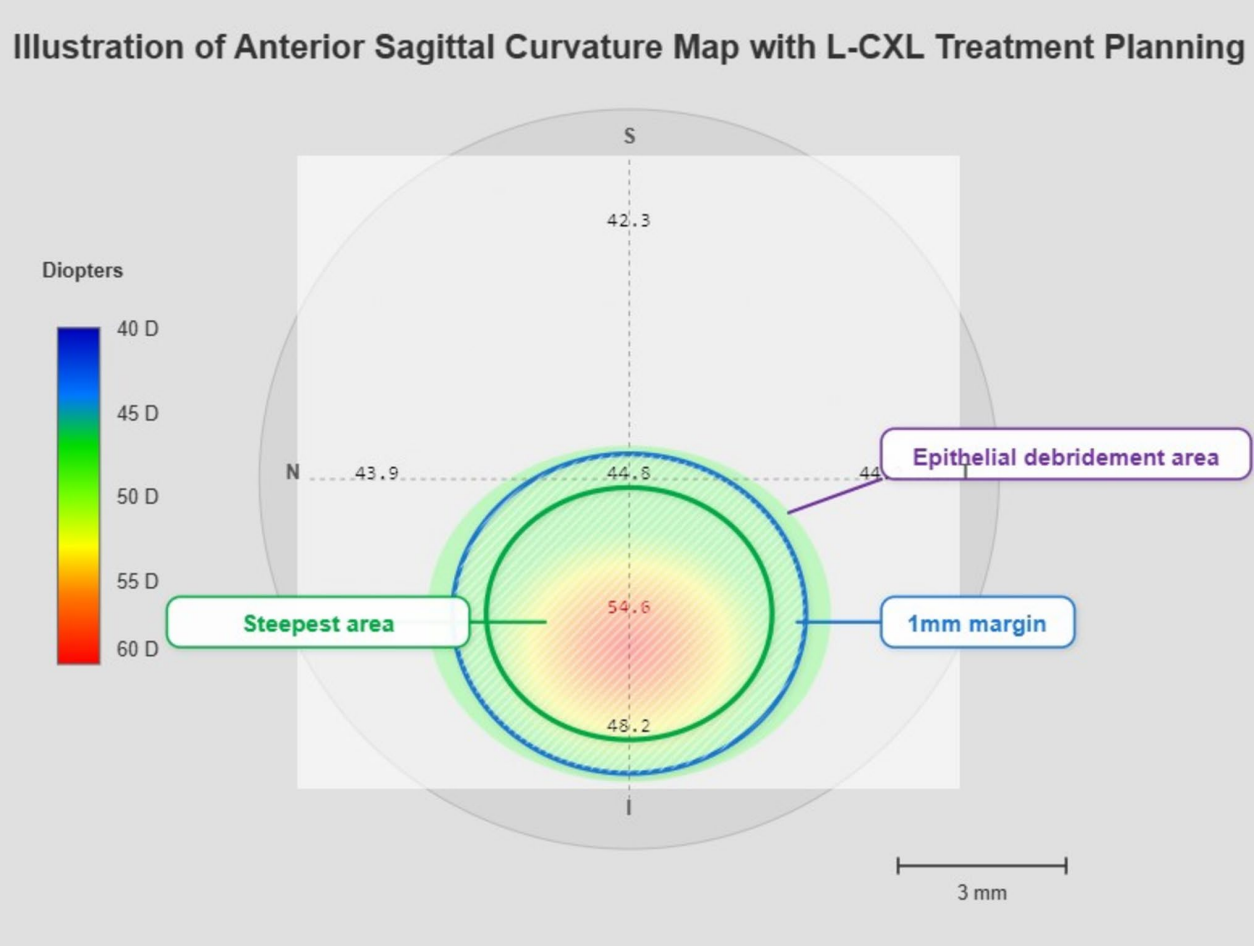
Illustration of pre operative anterior sagittal curvature map with L-CXL treatment planning

Treatment Planning and Protocol Cone-centered L-CXL was was performed under local anesthesia when possible. The area of treatment was determined by a team of senior corneal specialists based on the clinical exam and imaging. The cone-centered treatment area was determined based on anterior sagittal curvature maps from the Sirius tomographer. The treatment zone encompassed the steepest area of the cone plus a 1 mm surrounding margin to ensure complete coverage of the biomechanically weakened area (Fig. 1). This approach was chosen to concentrate the crosslinking effect on the most affected region while sparing the relatively normal corneal tissue. The epithelium was debrided over the area planned to be treated, and riboflavin was applied every 2 min for 30 min. An accelerated protocol was performed using an illumination device with an intensity of 9 mW/cm2 (UV-XTM 2000; IROC Innocross Zug, Switzerland) for 10 min applied over the area of de-epithelialization. Riboflavin continued to be applied every 2 min during UV-A exposure. The total UV-A exposure was calculated at 5.4 J/cm^2^ (9 mW/cm^2^ for 10 min).At the end of the procedure, the eye was irrigated with a balanced salt solution, and a bandage contact lens (Purevision, Bausch & Lomb, Inc.) was placed followed by the application of Dexamethasone Sodium Phosphate 0.1% and Moxifloxacin Hydrochloride 0.5%. The post-operative regimen included Moxifloxacin Hydrochloride 0.5% 4 times a day until the removal of the bandage contact lens, Loteprednol Etabonate 0.5% 4 times a day, tapered down over a month, and preservative free artificial tears were used as needed.

### Follow-up

Patients were examined 1 day post-operatively, 1 week (± 2 days) later for bandage contact lens removal as per corneal re-epithelialization, and at 1, 3, 6, and 12 months post-operatively. Manifest refraction, best corrected distance visual acuity (BDVA), and tomography were performed periodically in addition to the slit lamp examination starting at 3 months post-operatively.

### Data collection

At the first presentation, patients' demographics, clinical presentation, and tomographic imaging were analyzed. In addition, surgical data were retrieved, and clinical presentation and tomographic imaging were collected at 3 months, 6 months, and 12 months post.

### Statistical analysis

Statistical analyses was performed by IBM SPSS Statistics 24 (IBM, NY, United States). Distributions for different category parameters were measured, and matched pair analyses (small sample size) were carried out. A comparison of the changes in BDVA, K-max, and minimal pachymetry was performed with aparametric matched-pair analysis. An additional subgroup analysis was performed according to K-max, cylinder, and visual acuity—to evaluate the characteristics of improved patients and to assess which patients are most likely to benefit from the procedure. The overall significance level was set at an alpha of 0.05.

## Results

### Demographic and pre-operative data

The study identified twenty-four consecutive eyes of eighteen patients diagnosed with progressive keratoconus who underwent L-CXL between 2018–2021. The mean age at diagnosis of keratoconus was 22.5 ± 6.7 years (range, 14 to 41.3 years). Twelve patients (70.5%) were males. The baseline characteristics pre-L-CXL data, including the mean BDVA, cylinder, spherical equivalent (SEQ), K-max, and the minimal pachymetry (MP) are presented in Table 1.

**Table 1:** Baseline characteristics of patients and pre-operative data

Gender M (%)	74.2
Eye OD (%)	50
Age (y)	
Mean ± SD, Range [Min, Max]	22.7 ± 6.7, [14, 41.3]
Pre-operative BDVA (decimals)	
Mean ± SD, Range [Min, Max]	0.64 ± 0.2, [0.03, 1.0]
Pre-operative cylinder (D)	
Mean ± SD, Range [Min, Max]	−2.43 ± 1.5, [−5, −0.75]
Pre-operative SEQ (D)	
Mean ± SD, Range [Min, Max]	−2.33 ± 3.40, [−12, 2]
Pre-operative K-max (D)	
Mean ± SD, Range [Min, Max]	57.4 ± 6.0, [49.4, 78.9]
Pre-operative MP (µ)	
Mean ± SD, Range [Min, Max]	468.4 ± 39.0, [386, 552]

*M* male; *SD* standard deviation; *min* minimum; *max* maximum; *y* years; *D* diopters; *SEQ* spherical equivalent; *BDVA* best corrected distance visual acuity; *MP* minimal pachymetry

### Characteristics following L-CXL

The mean follow-up time for patients was 17.95 ± 7.7 months. No intraoperative complications occurred. Postoperatively, mild haze was observed in one eye, dry eye syndrome in eleven eyes, and papillae in three eyes.

**Fig. 2:**
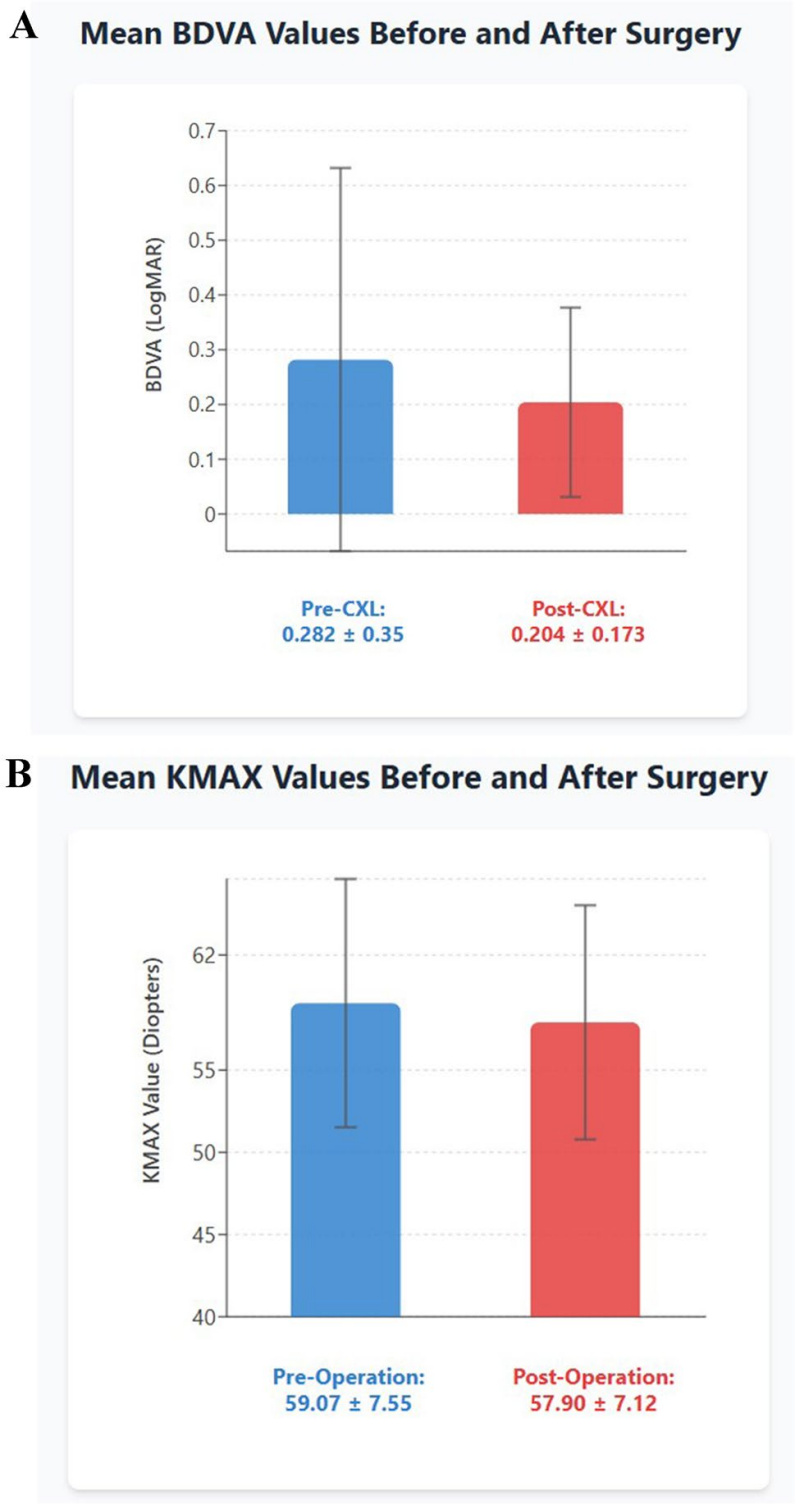
Preoperative and 12 months post L-CXL BDVA (**A**) and Kmax (**B**) parameters

All three visual and refractive parameters, BDVA,K-max and cylinder values, remained stable at 12-month postoperatively. A small reduction in MP from baseline values of 460.0 ± 41.7 µ to 451.4 ± 43.0 µ at 12 months after L-CXL (*P* < 0.001) was noted. Figure 2 shows preoperative and 12 months post CXL average BDVA parameters, and Kmax parameters.

### Best distance corrected visual acuity

**Fig. 3:**
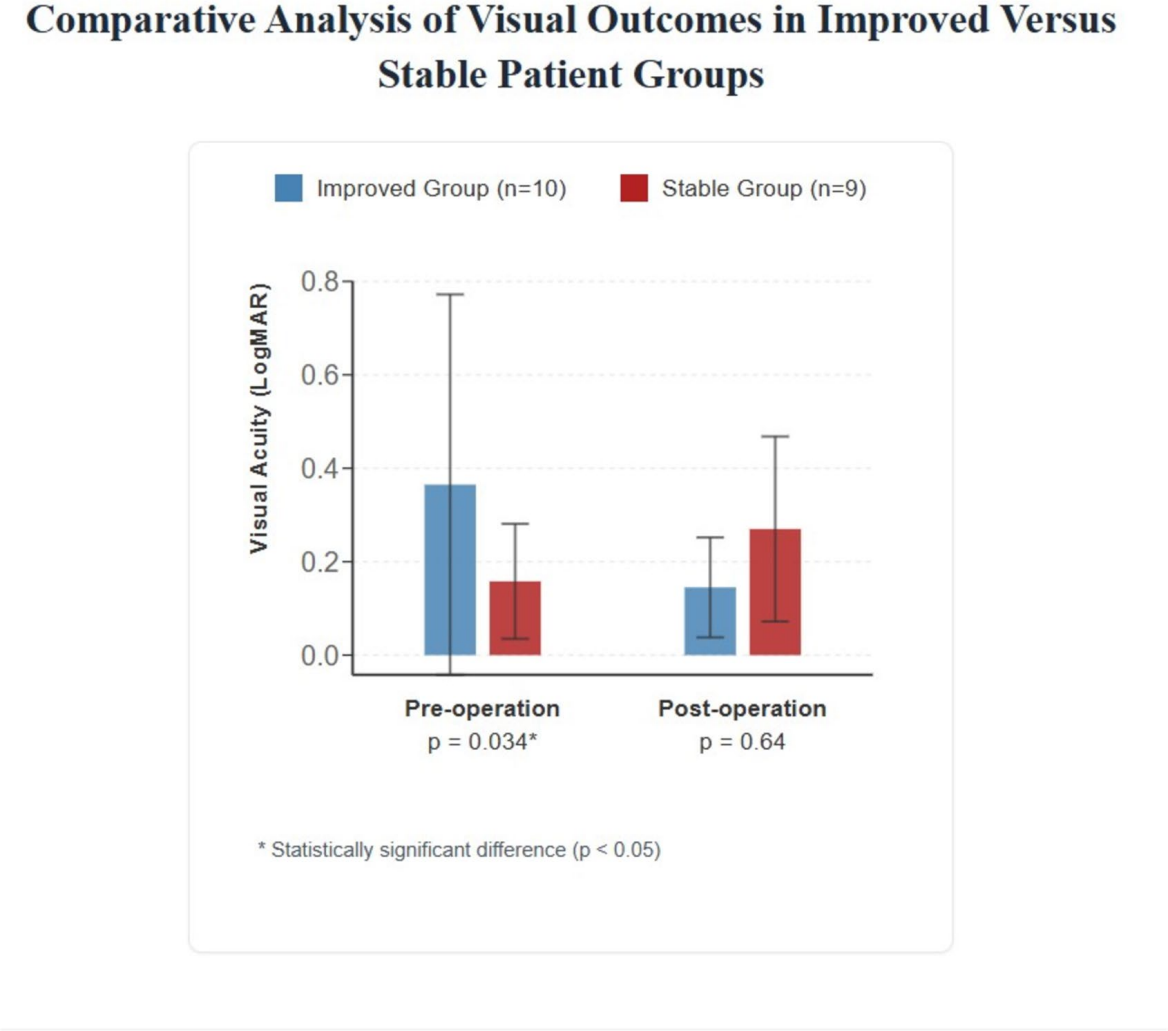
Pre and 12 months post LCXL average parameters in the stable visual acuity group compared to the improved VA group

The average BDVA before CXL was 0.282 ± 0.35 LogMar, remained overall stable at 12-month post-surgery at 0.204 ± 0.173 LogMar (*P* = 0.395) (Fig. 2A). However, ten out of the twenty-four eyes (42%) of 8 patients demonstrated an improvement of 1–4 lines in BDVA at 12 months of follow-up. Figure 3 shows preoperative and 12 months postoperative parameters in this group. The remaining eyes were all stable, and no one lost BDVA. Analysis of the improving eyes showed a significantly lower initial preoperative BDVA than the stable eyes (*P* = 0.034), and they achieved a similar BDVA at 12 months (*p* = 0.64). Other baseline characteristics were similar between these two groups, such as age (*p* = 0.302), gender (*p* = 0.93) and Kmax (*p* = 0.35).

### K-max

Average K-max remained stable at 12-month postoperatively (Pre op Kmax = 59.07 ± 7.55, and Post-op Kmax was 57.90 ± 7.12 *P* = 0.238) (Fig. 2B). However,13 out of the 24 eyes (54%) demonstrated an improvement in K-max at 12 months postoperatively of over 0.5 D. Six of these eyes had improvement in both BDVA and Kmax. None of the eyes developed an increase in Kmax throughout the follow-up.

**Fig. 4:**
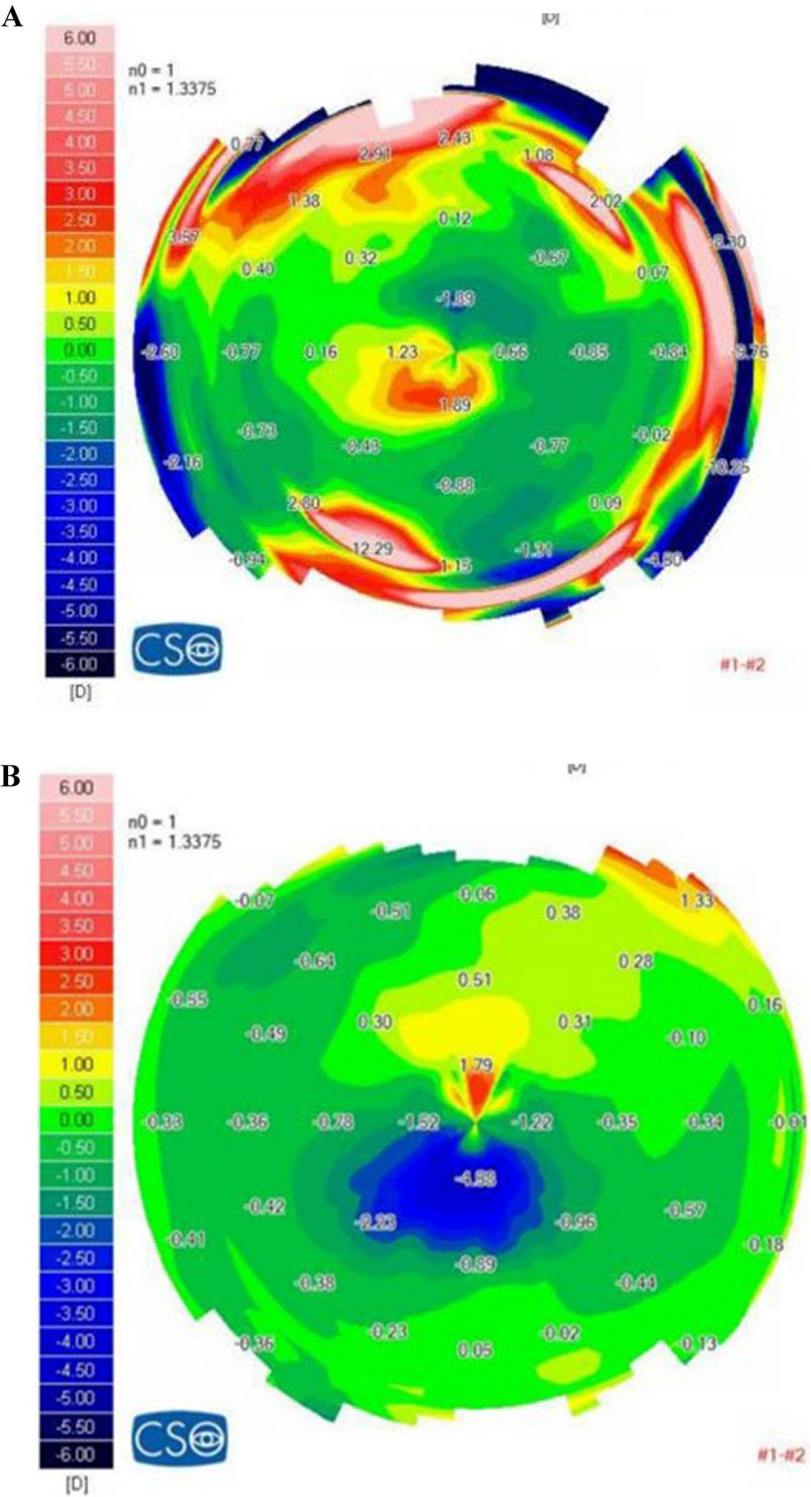
Difference map of the right eye sagittal anterior map showing the keratoconus progression prior to cone-centered L-CXL (**A**), and the keratoconus regression 2 years after cone-centered L-CXL (**B**)

Figure 4 demonstrates an example of keratoconus regression after cone-centered L-CXL. This patient's visual acuity and minimal pachymetry remained stable at 0.174 LogMARand 490 µm before and after the L-CXL. The other eye of the same patient was not treated and slowly progressed in Kmax values from 49.13D to 50.05D in almost 3 years of follow-up.

### Refractive cylinder

The refractive cylinder values remained overall stable at 12-month post-surgery (*P* = 0.326). However, six out of the twenty-four eyes (25%) of six patients demonstrated an improvement in the refractive cylinder at 12 months of follow-up. The initial pre-operative K-max of patients in this improved group was significantly lower than that of patients in the stable group, 55.7 ± 3.3 D, vs. 56.7 ± 6.4 respectively (*P* = 0.039).

## Discussion

In this study, we reviewed the efficacy of the cone-centered L-CXL procedure using a standard CXL device. We demonstrated that L-CXL even without eye tracking, is successful not only in stabilizing the keratoconus but even resulted in an improvement in BDVA and Kmax in 42% of the eyes. This was achieved utilizing the standard and accessible CXL platform and just modifying the illumination exposure technique on the affected area without treating the unaffected cornea. The procedure was safe, with no evidence of progression in any of the eyes.

It was also previously published that VA improves after CXL ([11]. Zarei-Ghanavati et al. [11] showed improvement of BDVA from a mean preoperative of 0.26 ± 0.029 logMAR. to 0.17 ± 0.014 logMAR, 8 months post operatively in all 22 eyes. We found that patients who improved their BDVA had significantly lower pre-operative visual acuity compared to stable BDVA patients, suggesting that these patients are better targeted for the treatment. In summary, patients with lower BDVA, who are sometimes referred to as more complicated patients, may benefit more from a customized procedure safely.

As shown in the recently published 15-year results of the CXL procedure [12], it is an effective method in the treatment of keratoconic eyes in the progressive stage of the disease and achieved long-term stabilization without serious complications. CXL was one of the more promising developments of the previous decade, in the management of keratoconus, and L-CXL could be of the promising procedures of the present. The focal irradiation results in localized changes to the corneal biomechanics and flattening [13]. The collagen fibril orientation in keratoconus is disrupted, especially in the region of the cone, and this area was shown to favor a reduced local elastic modulus [14]. Sinha Roy et al. [6] conducted a computational modeling study demonstrating that smaller, cone-centered CXL treatments achieve greater curvature reductions compared to broader treatments, supporting the biomechanical rationale for customized approaches. The work analyzed four customized CXL methods in simulated 50-eye models to establish this principle.

Studies with previously available devices have shown that localized CXL with eye tracking is as safe and effective as the standard procedure for stopping keratoconus progression [15]. Seiler et al. [16] discovered that the change in K-max was greater in the L-CXL group using the Avedro eye tracking system. Shetty et al. [17] found that a ring tangential map protocol using the Avedro KXL system provided the greatest decrease in curvature and greatest improvement in visual acuity per unit energy dose to the cornea, compared with a uniform treatment, a sector axial map protocol, and a ring axial map protocol. The previously available KXL II system with real-time eye tracking has also shown subjective improvement in patients [18, 19]. Mazzotta et al. used a customized CXL transepithelial approach with intraoperative supplemental oxygen and demonstrated a significant improvement in corneal curvature and BDVA in 27 eyes [20]. These studies demonstrate promising results for L-CXL using the Avedro L-CXL device, yet since the company (now called Glaukos) has discontinued distribution of these devices, thus they are not currently available. Our study demonstrated that performing L-CXL with a standard device was safe and effective, comparable to advanced devices mentioned, and is readily available in most centers. Our approach is more accessible to a broader range of practitioners, especially in small centers with no available sophisticated devices.

Different L-CXL protocols have gained added attention recently; Seiler et al. employed L-CXL with customized maximal irradiation ranging from 5.4 J/cm2 up to 10 J/cm2, centered on the maximum posterior elevation, and found improved maximal flattening [16]. Cassagne et al. performed topography-guided CXL in zonal patterns with total irradiance ranging from 5.4 J/cm2 in regions surrounding the cone to 15 J/cm2 on top of the cone using 30 mW/cm2 pulsed UV-A and found significant improvements in CDVA, K-max, and mean keratometry in the inferior part of the cornea [7]. Nordström et al. found improved VA, and K-max in eyes that underwent asymmetrical treatment centered on the area of maximum corneal steepness, with treatment energies ranging from 7.2 J/cm2 to 15.0 J/cm2 [21]. The protocols mentioned above use a higher intensity illumination device in comparison to the standard illumination intensity used in our study (9 mW/cm2) based on the general concept that applying greater irradiation leads to greater stiffness in the maximal cone region and less in surrounding regions. It is of note that higher illumination intensity might have a higher flattening effect, but it might not be necessary due to the stability of the patients in our study. Future studies exploring higher fluence protocols, and their safety and effectiveness with standard devices is important.

Webb et al. [9] demonstrated in 10 porcine eyes post L-CXL, utilizing Brillouin microscopy, that the three-dimensional biomechanical properties (stiffening effect) of CXL extends beyond the irradiated area, with a broad transition zone of approximately 600 μm between the fully crosslinked and non-crosslinked sections. Thus, even a good stiffening effect in the specific keratoconus treated area has a biomechanical impact on the transition zone as well. Our study highlights the concept that cone-centered L-CXL using standard devices, physicians across the globe may achieve promising results.

The limitations of our study are the retrospective nature and a small cohort. Moreover, a multicenter, multinational prospective study that enables studying different populations across the world is necessary to achieve higher external validity. An additional limitation of this study is that we did not systematically measure corneal demarcation lines postoperatively which could have provided information about the depth and extent of the crosslinking effect in the localized treatment areas.

In conclusion, this study demonstrates that cone-centered L-CXL utilizing a standard CXL device is a successful method for stabilizing the progression of keratoconus and improving vision in almost half of the eyes. Our study provides clinical validation of real-life experience of this CUSTOMIZED CXL concept using a conventional device. We show in this study that even more advanced eyes, with worse VA, may benefit from customized procedure,
